# Effects of a Pseudophysiological Environment on the Elastic and Viscoelastic Properties of Collagen Gels

**DOI:** 10.1155/2012/319290

**Published:** 2012-07-12

**Authors:** Sébastien Meghezi, Frédéric Couet, Pascale Chevallier, Diego Mantovani

**Affiliations:** ^1^Laboratory for Biomaterials and Bioengineering, Department of Mining Metallurgy and Materials Engineering and University Hospital Research Center, Laval University, Quebec City, QC, Canada G1V 0A6; ^2^Laboratory for Biomaterials and Bioengineering, Laval University, Pavillon Pouliot, 1745-E, Québec City, QC, Canada G1K 7P4

## Abstract

Vascular tissue engineering focuses on the replacement of diseased small-diameter blood vessels with a diameter less than 6 mm for which adequate substitutes still do not exist. One approach to vascular tissue engineering is to culture vascular cells on a scaffold in a bioreactor. The bioreactor establishes pseudophysiological conditions for culture (medium culture, 37°C, mechanical stimulation). Collagen gels are widely used as scaffolds for tissue regeneration due to their biological properties; however, they exhibit low mechanical properties. Mechanical characterization of these scaffolds requires establishing the conditions of testing in regard to the conditions set in the bioreactor. The effects of different parameters used during mechanical testing on the collagen gels were evaluated in terms of mechanical and viscoelastic properties. Thus, a factorial experiment was adopted, and three relevant factors were considered: temperature (23°C or 37°C), hydration (aqueous saline solution or air), and mechanical preconditioning (with or without). Statistical analyses showed significant effects of these factors on the mechanical properties which were assessed by tensile tests as well as stress relaxation tests. The last tests provide a more consistent understanding of the gels' viscoelastic properties. Therefore, performing mechanical analyses on hydrogels requires setting an adequate environment in terms of temperature and aqueous saline solution as well as choosing the adequate test.

## 1. Introduction

Cardiovascular diseases are one of the main causes of death all around the world [1]. Functional vascular tissue engineering (VTE) aims to produce a functional blood vessel to replace diseased arteries. A common workflow for the maturation process of tissue-engineered blood vessels includes the following steps: scaffold preparation, cell seeding, and maturation in a bioreactor [2, 3].

Within this work, collagen type I hydrogels have been selected as suitable scaffolds for vascular tissue engineering. Collagen is one of the main components of a blood vessel's extracellular matrix. Its unique biological properties such as its nontoxicity, low immunogenicity, and antigenicity make it a suitable scaffold material to promote vascular smooth muscle and endothelial cell adhesion and proliferation [4–6].

Since the initial mechanical properties of collagen gels are very low, maturation of constructs (scaffolds seeded with cells) in a controlled environment is required to produce a mechanocompatible tissue [7]. This environment must respect cell culture constraints in terms of temperature and the biochemical and mechanical environment. This specific environment may be different from the environmental conditions used for preparation and conservation of scaffolds prior to cell seeding and culture in a bioreactor. In order to optimize the process of developing tissue-engineered blood vessels—from the initial step of scaffold preparation through the final steps involving tissue maturation under mechanical stimulation—it is important to measure the mechanical properties of the material throughout the procedure. No normative regulates the mechanical characterization of hydrogels in the perspective of tissue regeneration or biomedical applications. In this context, many different protocols can be referenced even concerning the mechanical characterization of a specific material such as collagen. Mechanical conditioning (strain amplitude, strain rate, number of cycles), temperature (room temperature or body temperature), strain rate during the test, and surrounding environment are some examples of the parameters that can differ from one study to another (Table 1).

The aim of this work is to evaluate the effects of different environments on the elastic and viscoelastic properties of collagen gels, in order to determine the optimal environmental conditions for mechanical characterization of the scaffold prior to maturation in a tissue bioreactor. Therefore, in this study, the strain rate applied during the mechanical and viscoelastic characterization was chosen according to the strain rate estimated in a bioreactor for VTE. This study focuses on three factors that are considered to have an impact on the mechanical state of collagen scaffolds: temperature (T), hydration (H), and mechanical preconditioning (PC). The factor hydration refers to both the aqueous environment and the ionic strength of the solution.

Temperature drives the molecular mobility of polymer chains and can break weak interactions such as hydrogen bonds. Consequently, with an increased temperature, the viscosity response of the material is less solicited [15, 16]. The hydrated environment plays a major role on the instantaneous and viscous behaviors of hydrogels [17]. First, in an aqueous saline environment, the viscous part of the material is favored because of the plasticizing effect of water [18]. Second, the change in ionic strength, because of the saline solution, will affect the molecular interactions within the collagen gel  [19]. Mechanical preconditioning is also an important parameter when considering the characterization of mechanical properties of living tissues. Fung was the first to introduce this consideration [20]. Indeed, mechanical preconditioning is believed to remove tissue stresses and strains history, so as to obtain a repeatable stress-strain relationship.

In this study, biomechanical characterizations were conducted either at room temperature or at physiological temperature (37°C), in air or in an aqueous saline solution (phosphate-buffered saline solution—PBS), and either after or not after mechanical preconditioning. The effects of these parameters and their eventual interactions on the mechanical and viscoelastic properties were evaluated and compared with the pseudophysiological conditions established in the bioreactor.

## 2. Materials and Methods

### 2.1. Sample Preparation

Type I collagen was extracted from rat-tail tendons and solubilized in acetic acid solution (0.02 N) at a concentration of 4 g/L in accordance with a protocol previously described [21]. The collagen solution (2 g/L) was then mixed with Dulbecco's modified Eagle medium (DMEM, Gibco, Invitrogen Corporation, Burlington, ON, Canada, 1.1X), NaOH (15 mM), and HEPES (20 mM) in deionized water. Finally, this mixture was poured into moulds and let jellify overnight at 4°C. 

### 2.2. Mechanical Characterization

**Figure 1:**
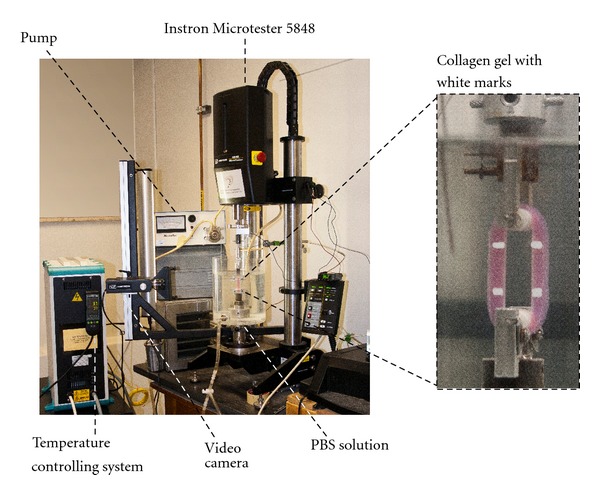
Experimental setup for tensile and relaxation tests on collagen gels. White marks were applied on the gels in order to observe the strain of the sample during testing.

**Figure 2:**
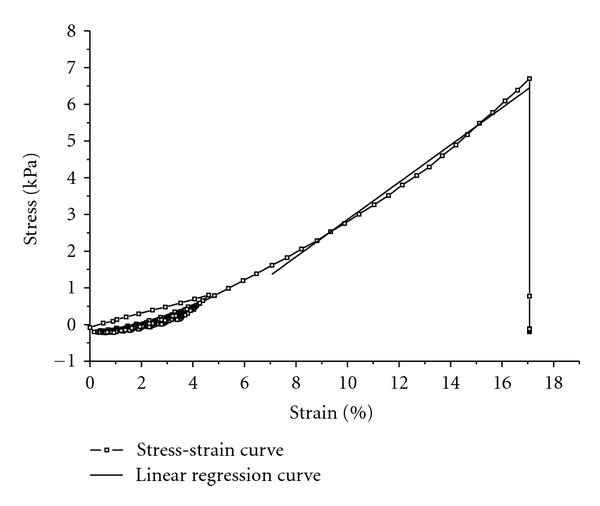
Example of a stress-strain curve obtained from a tensile test on a preconditioned collagen gel (10 cycles of loading and unloading) in air at 23°C. Linear modulus was calculated from the slope of the linear regression. The linear regression was determined by successively adding points in data in the left direction (starting from the point at rupture) as long as the squared *R* was >0.990.

Tensile tests were carried out on toroïdal-shaped collagen gels (dimensions of the two squared cross-sections: 5 mm × 6 mm, ID = 22 mm) with an Instron 5848 MicroTester (Instron Corporation, Norwood, MA, USA). White marks were drawn on the gels to measure the strain with a video camera as a function of the applied load (Figure 1). In order to avoid any damage to the samples before testing, gels were removed from their moulds and put in a phosphate-buffered saline solution (PBS 1X, Fischer Scientific, Ottawa, ON, Canada) where the fixation to the supports was directly carried out. The dimensions of the cross-sections were then measured. After 5 minutes of environmental conditioning, mechanical tests were performed either in a bath containing PBS solution or in air and either at 23°C or at 37°C (Figure 1). Samples tested in air at 37°C required an environmental chamber (series 3119, Instron Corporation, Norwood, MA, USA) adapted to the Instron 5848 MicroTester. Samples were mechanically preconditioned to 5% strain or not preconditioned at all. Mechanical preconditioning consisted of 10 cycles of loading and unloading at 5% strain and a 1% s^−1^ strain rate. Then samples were tensile-tested until failure at a strain rate of 5% s^−1^. This value was chosen after an estimation considering the flow, the pressure, and the pulsation of medium culture that characterize the mechanical constraints in the bioreactor. Stress-strain curves were thus obtained. The tensile linear modulus is calculated by computing the slope of the linear region of the stress-strain curve before break [19], as shown in Figure 2.

### 2.3. Stress Relaxation Test

**Figure 3:**
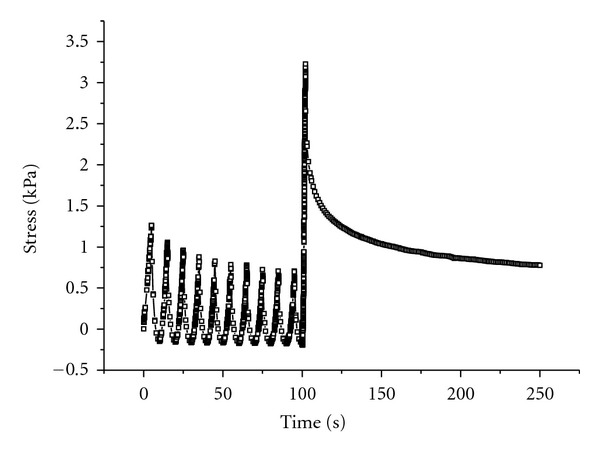
Example of a curve obtained from a stress relaxation test on a preconditioned collagen gel in air at 23°C.

Samples were prepared in the same conditions as for tensile tests. Similarly to tensile tests, relaxation tests were performed in a bath containing PBS solution or in air and at 23°C or at 37°C. Prior to the test, samples were mechanically preconditioned to 5% strain (1% s^−1^, 10 cycles) or not. The relaxation test consisted of stretching the sample at a 10% strain (5% s^−1^) and maintaining the strain constant for 150 s. The stress was recorded as a function of time as shown in Figure 3. This figure confirms the consistency in setting the parameters of mechanical preconditioning since the decline in the maximum stress at each cycle reached an equilibrium state [20].

### 2.4. Design of Experiment and Statistical Analysis

A factorial experiment was designed in order to evaluate the individual effect of three factors (temperature, hydration, and mechanical preconditioning) on the mechanical and viscoelastic properties of collagen gels. Each factor had two levels: temperature (23°C or 37°C), hydration (PBS or no PBS), and mechanical preconditioning (PC or no PC). Therefore, this factorial experiment is constituted of 8 conditions of testing (Table 2). For each condition of each characterization, 5 to 8 samples were analyzed. In order to consider the intrinsic variability of the samples and the random effect of the day of preparation, a linear mixed model was chosen for the statistical analysis of the data. Statistical analysis was performed with the open source software “R” [22].

## 3. Results and Discussion

### 3.1. Mechanical Characterization

**Figure 4:**
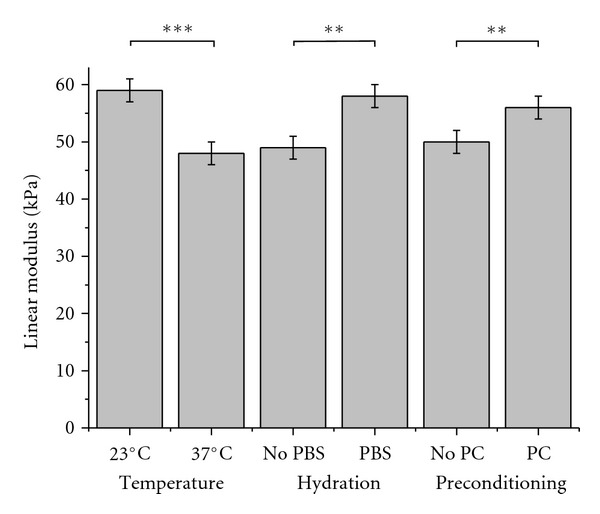
Tensile linear modulus of ring-shaped collagen gels as a function of temperature (experiments performed either at 23°C or 37°C), hydration (either with or without a PBS solution), and mechanical preconditioning (either with or without mechanical preconditioning PC). Values are expressed as mean ± standard error. Significance: *P < 0.05; **P < 0.01; ***P < 0.001.

The effects of temperature, hydration, and mechanical preconditioning on the linear modulus of collagen gels are shown in Figure 4. These environmental factors have a significant impact on the linear modulus. Indeed, performing the tests at 37°C decreased the values of the linear modulus from 59 ± 2 kPa when done at 23°C to 48 ± 2 kPa, corresponding to a 19% decrease (*P* < 0.001). The opposite effect was noticed for the tests done either in PBS solution or in air: the linear modulus increased from 49 ± 2 kPa in air to 58 ± 2 kPa in PBS solution, corresponding to an 18% increase (*P* < 0.05). Finally, mechanical preconditioning also led to a slight increase of the linear modulus. It is worth noting that mechanical preconditioning did not improve the reproducibility of the measurements since the variances are quite similar with or without the precycles prior to the test (data not shown).

These results evidenced that temperature and hydration are critical parameters for the estimation of a linear modulus from tensile tests. Consequently, performing tensile tests in different environments can result in great differences in the estimation of the stiffness of the gels. For example, the linear modulus is 46 ± 1 kPa when measured in PBS solution at 37°C, whereas this value is 59 ± 3 kPa when the test is done in PBS solution but at 23°C, which represents a difference of 28% on the linear modulus estimation. Therefore, in the perspective of using the collagen scaffold in the bioreactor, the pseudophysiological conditions will be referred to as the aqueous saline solution at 37°C without mechanical preconditioning. For a better understanding of the mechanisms involved as a function of the conditions of testing, it is preferable to study the influence of these environmental factors on the viscoelastic parameters of the gels. This will allow anticipation of the behavior of the collagen scaffold subdued to mechanical constraints inside the bioreactor.

### 3.2. Stress Relaxation Test

**Figure 5:**
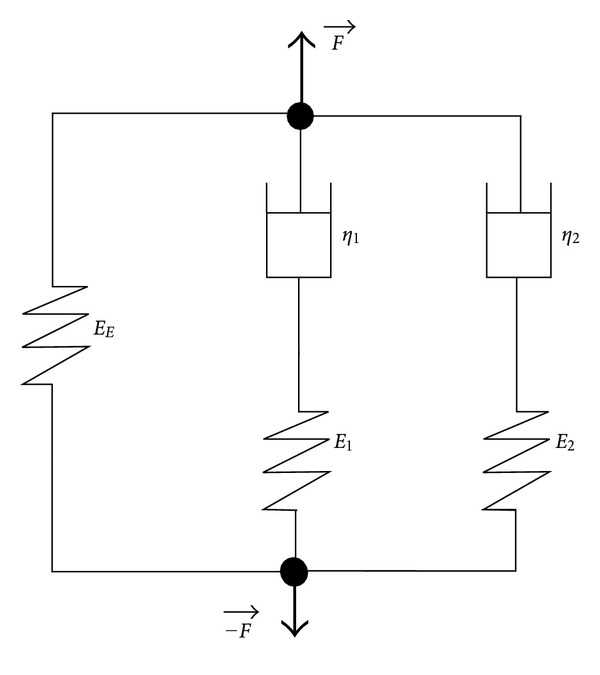
Viscoelastic model constituted of a spring associated in parallel with two Maxwell elements. A tensile force *F* is applied to this model. *E*_*E*_, *η*_*I*_, and *E*_*i*_ are, respectively, the elastic modulus, the viscosity, and the relaxation moduli associated with the model.

**Figure 6:**
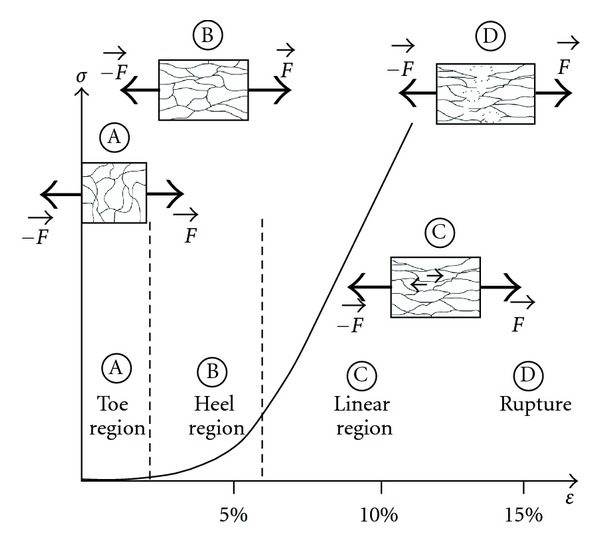
Proposed molecular mechanism involved during the collagen gel stretching when applying a tensile load *F*. Four regions can be identified: (A) “toe region” (low strains), (B) “heel region” (nonlinear region), (C) linear region (small arrows symbolize friction), and (D) rupture of the gel.

Stress relaxation tests can provide information on how the studied factors influence the viscoelastic properties of the hydrogels in the long term (e.g., for a period of time greater than a few seconds). The relaxation processes are related to the physicochemistry of the stretched polymer, which can include interactions between the polymer chains and types of side chains, as well as length and diameter of the polymer chains. A relaxation time is related to each one of these processes. Considering that the collagen gel is constituted of fibrils with a range of lengths, multiple relaxation times are expected. The Weichert model has been previously used to explain stress relaxation of collagen gels [23]. It can be represented by a spring (elastic modulus of the spring *E*_*E*_) and Maxwell elements associated in parallel. Each Maxwell element *i* is composed of a spring (modulus *E*_*i*_) and a dashpot (viscosity *η*_*i*_) associated in series (Figure 5). Empirically, the present model composed of two Maxwell elements was sufficient to fit these different relaxation times. Therefore, the relaxation modulus *E*_*R*_ (in kPa) can be expressed by the following expression:

(1) ER(t)=EE+E1×exp⁡(−tτ1)+E2×exp⁡(−tτ2),

where *E*_*E*_ and *E*_*i*_ (*i* = 1 to 2, in kPa) are, respectively, the elastic and the viscous moduli and *τ*_*i*_ are the relaxation times (*i* = 1 to 2, in s). The viscosity *η*_*i*_ of each Maxwell element can be calculated according to the following equation: *η*_*i*_ = *E*_*i*_ · *τ*_*i*_ in kPa*·*s. When the relaxation terms are ordered starting from the highest relaxation time to the smallest one (*τ*_1_ > *τ*_2_), the viscous moduli are ordered starting from the smallest to the highest one (*E*_1_ < *E*_2_), which is consistent with the fact that the main relaxation processes occur very early during the relaxation as it can be observed in Figure 3. A molecular mechanism of the origin of the viscoelastic processes is proposed in Figure 6. At low strains (region A), the collagen gel is composed of randomly oriented fibrils. In this region (the so-called “toe region”), crimps in the collagen gel are being removed at the fibrillar and then at the molecular level. The gel behaves as an uncoiling spring. The stiffness of the material in this region corresponds to the elastic modulus *E*_*E*_. In region B (the so-called “heel region”), collagen fibrils progressively become oriented in the direction of the tensile force $\vec{F}$ . In region C, the maximal extension of collagen fibrils is reached. Collagen fibrils slip past each other generating friction responsible for the viscous response [8, 24]. If we simplify the system and consider the length of collagen fibrils as a unique parameter of the relaxation processes, two main characteristic lengths (“shorter chains” and “longer chains”) would generate two different relaxation processes with their own amplitude *E*_*i*_ and relaxation time *τ*_*i*_. These two factors, amplitude and relaxation time, would both take place from the beginning of the relaxation, but the viscous process generated by friction between “shorter chains” will be rapidly negligible compared to those induced by “longer chains.” In order to understand the effect of an environmental factor on the viscoelastic properties of the gel, its influence on the molecular interactions between the collagen fibrils should be considered. Hence, any factor that will increase the molecular interactions between the collagen fibrils will induce an increase in *E*_*i*_ and *τ*_*i*_ because of the increased friction. This would lead to a more viscous response of the material to a mechanical stress.

#### 3.2.1. Effect of the Environmental Factors on the Viscosities

**Figure 7:**
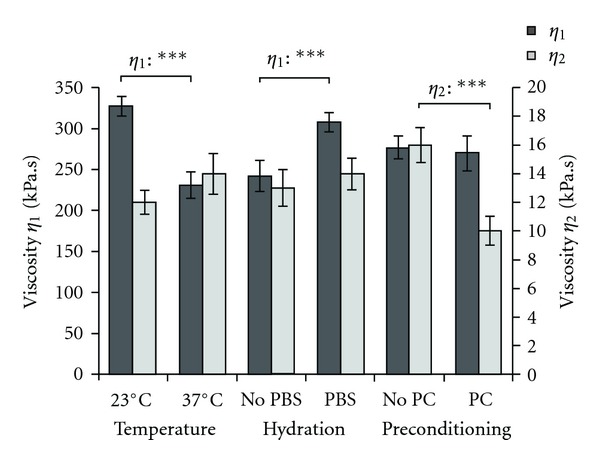
Viscosities of ring-shaped collagen gels as a function of temperature (experiments performed either at 23°C or 37°C), hydration (either with or without a PBS solution), and mechanical preconditioning (either with or without mechanical preconditioning PC). Viscosities *η*_1_ and *η*_2_ are extracted from the first and the second term of the second-decay exponential regression of stress-strain relaxation curves obtained on collagen gels. Values are expressed as mean ± standard error. Significance: *P < 0.05; **P < 0.01; ***P < 0.001.

Viscosities of the gels *η*_*i*_ related to each relaxation process were calculated from the values of the relaxation times *τ*_*i*_ and the viscous moduli *E*_*i*_ (Figure 7). Temperature drastically influenced the viscosity of the gels. Hence, *η*_1_ decreased from 327 ± 12 kPa*·*s, when measured at 23°C, to 231 ± 16 kPa*·*s when the test was performed at 37°C, respectively (*P* < 0.001). This effect could be explained by the fact that collagen fibrils offer less resistance to tension when temperature is increased due to two main phenomena: a higher molecular mobility at 37°C compared to 23°C and the possible scission of intramolecular links between collagen chains, such as hydrogen bonds or Van der Walls interactions [16, 25].

Concerning the influence of PBS solution, two processes have to be considered: the plasticizing effect of water and the ionic strength of the solution. Because of a high dipolar moment, water molecules break intra- and intermolecular dipole-dipole interactions as well as hydrogen bonding between protein molecules, creating hydrogen bonds with polar and charged groups such as –NH_2_, –OH, –COOH. Therefore, water molecules increase chain mobility within the biopolymer, decreasing its viscosity and stiffness [18]. The ionic strength has an opposite effect on chain motion. The ionic strength of PBS solution (1X, IS = 171 mM) is lower than the one of the gels (IS > 280 mM); thus, the ionic strength of the gels in this saline solution may be lowered because of the osmotic pressure that controls water in the gels. Electrostatic forces Δ*F*_el_ are closely related to ionic strength IS and increase when IS decreases (Δ*F*_el_ ~ exp⁡⁡(−IS^1/2^) [26]. As a consequence, if IS decreases, electrostatic forces are stronger, the gels are more viscous, and therefore become stiffer. Hence, *η*_1_ increased from 242 ± 19 kPa*·*s when measured in air to 308 ± 12 kPa*·*s when the test was performed in PBS solution, respectively (*P* < 0.001). This result evidences that the change in IS has a more important effect on the viscosity of the gels than the plasticizing effect of water.

Interestingly, mechanical preconditioning had a significant effect on *η*_2_ since this parameter decreased from 16 ± 1 kPa*·*s to 10 ± 1 kPa*·*s when the test was performed without and with mechanical preconditioning, respectively (*P* < 0.001). After mechanical preconditioning, collagen molecules are preferentially aligned in the direction of the strength and in a stretched configuration. This is schematically represented by the states B and C on Figure 6 where molecular frictions between collagen fibrils contribute to the viscoelastic properties of the gels as they slip pass each other [27]. Mechanical preconditioning may enhance the chains motion by disrupting some molecular interactions. It should be noticed that only the viscosity extracted from the second term of (1) is affected by the mechanical preconditioning. As discussed previously, the second term in the regression may be related to the more rapid relaxation processes involving “shorter” collagen fibrils. As a consequence, mechanical preconditioning may generate shorter polymer chains as a result of premature failure of collagen fibrils.

#### 3.2.2. Effect of the Environmental Factors on the Relaxation Times

**Figure 8:**
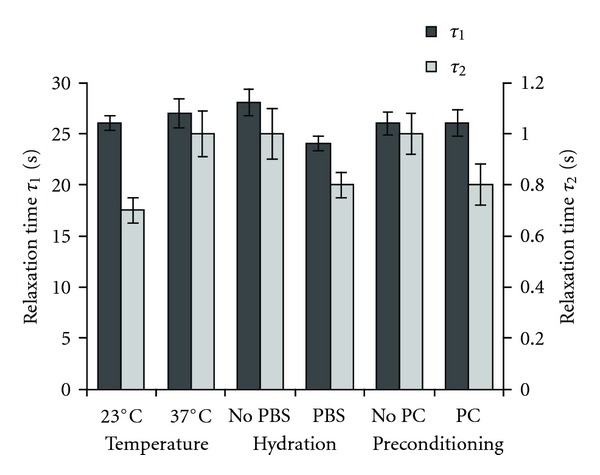
Relaxation times of ring-shaped collagen gels as a function of temperature (experiments performed either at 23°C or 37°C), hydration (either with or without a PBS solution), and mechanical preconditioning (either with or without mechanical preconditioning PC). *τ*_1_ and *τ*_2_ are the relaxation times of the gels considered in the viscoelastic model. Values are expressed as mean ± standard error. Significance: *P < 0.05; **P < 0.01; ***P < 0.001.

The relaxation times obtained from the second-decay exponential regression were one magnitude in difference, with the first and second processes of the viscous response relaxing within 26 s and less than 1 s, respectively (Figure 8). Despite the changes in viscosity as a function of the conditions of testing, no impact of the environmental factors could be identified. Therefore, the study of the influence of these factors on the viscous moduli may allow a further understanding of the origin of these changes.

#### 3.2.3. Effect of the Environmental Factors on the Relaxation Moduli

**Figure 9:**
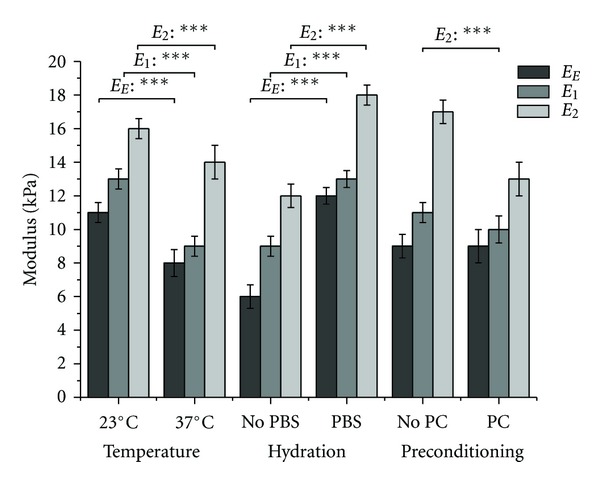
Relaxation moduli of ring-shaped collagen gels as a function of temperature (experiments performed either at 23°C or 37°C), hydration (either with or without a PBS solution), and mechanical preconditioning (either with or without mechanical preconditioning PC). *E*_*E*_, *E*_1_, and *E*_2_ are, respectively, the elastic and the viscous moduli of the gels considered in the viscoelastic model. The sum of all 3 moduli is the instantaneous linear modulus *E*_*R*0_ at a strain of 10%. Values are expressed as mean ± standard error. Significance: *P < 0.05; **P < 0.01; ***P < 0.001.

The effects of temperature, hydration, and mechanical conditioning on the elastic and viscous moduli are shown in Figure 9. An increase in temperature from 23°C to 37°C generated a decrease in *E*_1_ and *E*_2_ from 12.9 ± 0.6 kPa and 16.4 ± 0.6 kPa to 9.0 ± 0.6 kPa and 13.9 ± 1.0 kPa, respectively (*P* < 0.001). Performing the test in PBS resulted in a 44% and 50% increase in *E*_1_ and *E*_2_, respectively, versus performing the test in air (*P* < 0.001). Mechanical preconditioning decreased the value of *E*_2_ from 16.6 ± 0.6 kPa to 13.3 ± 0.8 kPa which is consistent with the decrease in *η*_2_ in the same conditions. Therefore, the environmental factors had an important influence on the viscous moduli explaining the variation of viscosity of the gels with the conditions of testing.

The same tendency is observed when considering the effects of temperature and hydration on the elastic modulus *E*_*E*_ (Figure 9). Indeed, *E*_*E*_ decreases from 11.1 ± 0.6 kPa when measured at 23°C to 7.8 ± 0.8 kPa when the test is performed at 37°C. Oppositely, *E*_*E*_ increases from 6.5 ± 0.5 kPa to 12.3 ± 0.7 kPa in air and in PBS solution, respectively. Consequently, stiffness of the gels is decreased with temperature whereas it is increased in the presence of PBS solution. As previously discussed, this can be explained by the fact that temperature increases molecular mobility and disrupts some weak molecular interactions. PBS solution has a lower ionic strength, which results in stronger electrostatic interactions lessening the molecular motion of collagen fibrils [26]. 

However, it should be noticed that the elastic modulus determined by relaxation tests (Figure 9) is different from the one determined by tensile tests (Figure 4). For example, in PBS solution at 37°C, the tensile linear modulus of collagen gels was 46 ± 1 kPa whereas the elastic modulus estimated by relaxation tests was 12 ± 1 kPa. This difference indicates that the linear modulus previously determined by tensile tests was not only related to the elastic behavior of the gels but also to the viscous response. A closer look at relaxation times clearly shows that most of the viscous processes relax after around 30 s (*τ*_1_) (Figure 8). Therefore, the rate of the ramp, 5% s^−1^, for stretching during tensile tests appears to be high. Nevertheless, this value was initially chosen in order to mimic the bioreactor conditions. In order to be sure that this material would withstand the mechanical constraints in the bioreactor without any rupture or plastic deformation, the elastic modulus needs to be considered. This is a crucial parameter to assess because it allows for the evaluation of the stiffness of the scaffold. The determination of the elastic modulus of the gels would require stretching the samples in their elastic range of strains, which is very narrow, or stretching them in quasi-static conditions which means at an infinitely low rate. In this context, relaxation tests are an interesting alternative to tensile tests regarding the estimation of the elastic modulus.

#### 3.2.4. Combined Effects of the Environmental Factors on the Viscoelastic Parameters

Statistical analyses allowed for the evaluation of the combined effects of the environmental factors and showed that the effects are magnified when two factors are combined. Indeed, the decrease in the elastic modulus with temperature is even more important when this factor is interacting with the variation of hydration (−55%, *P* < 0.05) or mechanical preconditioning (−70%, *P* < 0.01) whereas mechanical preconditioning alone has no notable effect on the viscoelastic parameters of the gels. Similarly, the decrease in viscosity with temperature is also more important when this factor is interacting with the variation of hydration (−66%, *P* < 0.01 for *η*_1_) or mechanical preconditioning (−66%, *P* < 0.01 for *η*_2_). Hence, *E*_2_ underwent a 53% decrease when measured in air at 23°C versus in PBS solution at 37°C. Performing the test at 23°C without mechanical preconditioning versus at 37°C with mechanical preconditioning caused a 75% decrease on *E*_2_ whereas temperature alone was responsible for a 15% decrease only (Figure 9). Therefore, mechanical preconditioning and aqueous saline solution appear to improve heat transfer facilitating the increase in molecular motion due to temperature.

## 4. Conclusion

The aim of this work was to evaluate the effects of temperature, hydration, and mechanical preconditioning on the elastic and viscoelastic properties of collagen gels. It was shown that all three factors have a significant effect on both elastic and viscous components of collagen gels' mechanical properties. Therefore, we conclude that mechanical tests of collagen scaffolds prior to their use and continuous evaluation in bioreactors should be tested in a pseudophysiological environment consisting of a bath with PBS solution at 37°C without any mechanical preconditioning.

Viscous processes are involved when tensile tests are performed at a strain rate representative of the mechanical stresses that are established in the bioreactor which impede the measurement of the elastic modulus. Consequently, relaxation tests appear to be more appropriate for the mechanical characterization of collagen gels in the perspective of their use for vascular tissue regeneration, since viscosity and viscous and elastic moduli can be accessed with this technique.

In the future, the preparation of collagen scaffolds should be optimized in order to reach appropriate mechanical properties assessed in the environment previously described. This optimization must not alter the biological performances of the scaffold. The pseudophysiological conditions will allow measuring the contribution of the cells to the overall mechanical properties of the constructs.

## Figures and Tables

**Table 1:** Example of various environments of mechanical characterization of collagen materials and tissues encountered in the literature [8–14].

Author	Materials	Experiments	Preconditioning/testing
Cornwell et al. [9]	Extruded type I collagen	(i) Uniaxial tensile tests	(i) Tests in air at RT
			(ii) No mechanical preconditioning
			(iii) *σ*_max⁡_ = *σ*_UTS_, rate: 0.83% s^−1^ (tensile tests)

Yang et al. [10]	Porcine esophagi	(i) Incremental stress relaxation tests	(i) Tests in air at RT
			(ii) No mechanical preconditioning
		(ii) Incremental cyclic tests	(iii) Rate: 0.83 mm/s
			(iv) Holding time: 300 s (relaxation tests)

Roeder et al. [8]	Type I collagen gels (0.3–3 mg/mL)	(i) Uniaxial tensile tests on dumbbell-shape samples	(i) Tests in a bath containing PBS at 37^°^C
			(ii) No mechanical preconditioning
			(iii) *σ*_max⁡_ = *σ*_UTS_, rate: 0.6% s^−1^ (tensile tests)

Feng et al. [11]	Type I collagen gels (1.67 mg/mL + 10^6^ cells/mL)	(i) Uniaxial tensile tests	(i) Tests in a bath containing medium culture at RT
		(ii) Stress relaxation tests	(ii) *σ*_max⁡_ = 33.3 kPa, rate: at 0.9% s^−1^ (×10, mechanical preconditioning)
		(iii) Creep tests	(iii) Holding time: 70 s (relaxation tests)
			(iv) *σ*_max⁡_ = *σ*_UTS_, rate: 0.9% s^−1^ (tensile tests)

Berglund et al. [12]	Type I collagen gels (2 mg/mL)	(i) Uniaxial tensile tests	(i) Tests in air at RT
		(ii) Stepwise stress relaxation tests	(ii) *σ*_max⁡_ = *σ* when *ε* = 0.2∗*ε*_UTS_; rate: 0.2 mm/s (×3, mechanical preconditioning)
		(iii) Creep tests	(iii) *σ*_max⁡_ = *σ*_UTS_, rate: 0.2 mm/s (tensile tests)

Assoul et al. [13]	Rat arteries	(i) Tensile tests on ring- and rectangular-shaped samples	(i) Tests in a bath containing PBS at 37^°^C
			(ii) No mechanical preconditioning
			(iii) *ε*_max⁡_ = 0.8–0.57, duration of the tests: 0.13–1 s (tensile tests consisting in one loading and one unloading)

Chan et al. [14]	Collagen membranes	(i) Uniaxial tensile tests	(i) Tests in air at RT
			(ii) No preconditioning
			(iii) *σ*_max⁡_ = *σ*_UTS_, rate: 0.083 mm/s (tensile test)

RT: room temperature; *σ*_max⁡_: maximal stress reached; *σ*_UTS_: stress at rupture; *ε*_UTS_: strain at rupture, *ε*_max⁡_: maximal strain reached.

**Table 2:** Design of the experiments. A complete factorial experiment was used to estimate the effects of temperature (T, 23 or 37^°^C), hydration (in a phosphate-buffered saline solution: PBS or without: no PBS) and mechanical preconditioning (C, with: PC or without: no PC) on the mechanical and viscoelastic properties of collagen gels.

Condition	Factor		
	T	H	C
1	23^°^C	No PBS	No PC
2	23^°^C	No PBS	PC
3	23^°^C	PBS	No PC
4	23^°^C	PBS	PC
5	37^°^C	No PBS	No PC
6	37^°^C	No PBS	PC
7	37^°^C	PBS	No PC
8	37^°^C	PBS	PC
